# Evaluation of the use of electronic medical record systems in Brazilian intensive care units

**DOI:** 10.5935/0103-507X.20180057

**Published:** 2018

**Authors:** José Colleti Junior, Alice Barone de Andrade, Werther Brunow de Carvalho

**Affiliations:** 1 Hospital Santa Catarina - São Paulo (SP), Brasil.; 2 Instituto da Criança, Hospital das Clínicas, Universidade de São Paulo - São Paulo (SP), Brasil.

**Keywords:** Electronic health records, Health information system, Health information technology, Medical informatics, Health surveys, Intensive care units, Brazil

## Abstract

**Objective:**

To examine the prevalence of the use of electronic medical record systems in Brazilian intensive care units and the perceptions of intensive care physicians regarding the contribution of electronic medical record systems toward improving safety and quality in clinical practice.

**Methods:**

Using an online questionnaire, physicians working in Brazilian intensive care units answered questions about the use of electronic medical record systems in the hospitals in which they worked. They were asked about the types of electronic medical record systems used and their levels of satisfaction with these systems in terms of improving quality and safety.

**Results:**

Of the 4,772 invitations sent, 204 physicians responded to the questionnaire. Most used electronic medical record and prescription systems (92.6%), worked in private hospitals (43.1%), worked in general adult intensive care units (66.7%) and used Private System A (39.2%); most systems had been used for between 2 and 4 years (25.5%). Furthermore, the majority (84.6%) believed that the electronic system provided better quality than a paper system, and 76.7% believed that electronic systems provided greater safety than paper systems.

**Conclusion:**

Electronic medical record systems seem to be widely used by the Brazilian intensive care physicians who responded to the questionnaire and, according to the data, seem to provide greater quality and safety than do paper records.

## INTRODUCTION

Information technology (IT) in healthcare is changing the way data are documented, stored, viewed, retrieved, shared, managed and consumed.^(1)^ Electronic health records have great potential in terms of improving health care, facilitating the rapid and accurate transmission of patient data, standardizing medical procedures, supporting decision making and allowing for the prevention of medical errors in real time.^(2)^ The use of IT in the health sector has been associated with improvements in safety and quality indicators, as well as cost optimization.^(2,3)^ A major transition is underway in patient-related data documentation with the adoption of electronic medical record systems (EMRSs).^(4)^

However, criticisms of the EMRSs that are currently available have been reported, including in regard to the alleged improvements in quality and patient safety.^(5)^ Some believe that the IT sector has not yet developed sufficiently adequate standards in this specific health field and has not achieved the necessary technological advances related to medical, nursing and multidisciplinary care that are required to establish a higher and standardized quality level.^(5)^

Nevertheless, across the world, more and more hospitals and health units have adopted EMRSs, including intensive care unit (ICU) settings, without knowing their actual impacts on the routines of this hospital sector. Some criticisms include that EMRSs absorb the medical and multidisciplinary team's time, thereby reducing their time at the patient's bedside.^(6-8)^ There are questions regarding the origin of EMRSs, as some emerged from commercial interests in improving hospital billing and were adapted for clinical use, while others were developed based on clinical applications. Some EMRSs interact with prescribers, warning of drug interactions and blocking incorrect administration routes, while others assist in inventory control or facilitate communication with clinical analysis laboratories and diagnostic imaging services. Others are less sophisticated and ultimately are not user-friendly; therefore, they place an extra workload on the prescriber. In this context, we do not know the reality of the use of EMRSs in Brazilian ICUs, as there are no specific studies on the subject.

The objectives of this study were therefore to examine the prevalence of the use of EMRSs in Brazilian ICUs and to evaluate intensive care physicians' perceptions of the EMRSs' contributions to improved safety and quality in clinical practice.

## METHODS

The present work is a cross-sectional, quantitative, descriptive and exploratory study that was carried out in collaboration with the *Associação de Medicina Intensiva Brasileira* (AMIB) using the AMIBnet platform. The study protocol was approved by the AMIB Fund board in 2016. The data were collected using an online questionnaire that was developed for investigations of the context and main characteristics of the use of electronic medical record and prescription systems in Brazilian ICUs.

The instrument was developed for a descriptive study and included seven multiple-choice questions and three questions with scores ranging from zero to ten. To compare the commercial EMRSs available in the market, the two main EMRSs were referred to as Private System A and Private System B, although respondents had access to the EMRSs' trade names. Once developed, the questionnaire was uploaded to an online platform (SurveyMonkey^®^) in order to facilitate access and increase the participation of physicians in the AMIB register from across the country.

The sample consisted of 204 physicians; the selection criterion was working in an ICU. Requests for participation in the study were made by sending links to the questionnaire via e-mail. Participation was linked to the Internet Protocol (IP) address of each computer system, and only one submission per IP address was allowed in order to prevent duplicate responses by the same physician.

The data collection period was from December 2016 to October 2017. Throughout this period, the e-mail link was available for access. The intensive care physicians registered with the AMIB received an initial e-mail that included an attached invitation letter from the principal investigator and a message about access to the online survey; it directed them to take the survey completely anonymously on the SurveyMonkey^®^ platform. A total of 4,772 invitation letters were sent out. Electronic responses were automatically archived in the platform's online database. Once the number of required responses was reached (initial requirement = 200 responses), the data collection phase was ended.

### Statistical analysis

The collected data were extracted from the online platform and entered into Microsoft Excel. Then, the generated database was encoded in order to be able to use a specific statistical program. The data were analyzed using Stata statistical software, version 12.0.

To describe the use of electronic medical records in the ICU, a descriptive analysis of the data was performed by calculating simple frequencies and proportions for the categorical variables. In addition, the questionnaires included questions about safety and quality for which the participants were asked to provide a score between 1 and 10; the higher the score was, the greater their satisfaction with the use of the EMRS. The mean scores for these criteria were used for the analysis, and a Likert scale was also established^(9)^ in which 1 - 2 points indicated being very dissatisfied; 3 - 4 indicated being dissatisfied; 5 - 6 indicated being neutral; 7-8 indicated being satisfied; and 9-10 indicated being very satisfied.

In addition, a bivariate analysis was performed to investigate any possible associations between the use of electronic medical records and the other variables. The Pearson Chi-square test was used to verify this association, and a significance level of 0.05 was established for the intergroup comparisons.

## RESULTS

The results of this study refer to 204 physicians working in the ICU. The questionnaire response rate was 4.3%. Among the survey respondents, 92.6% used electronic medical record and prescription systems (92.6%), 43.1% worked in private hospitals, 66.7% worked in general adult ICUs and 39.2% used Private System A, with a time of use of between 2 and 4 years (25.5%). Table 1 shows the general distribution and use of EMRSs among the participating physicians.

**Table 1:** Descriptive characteristics of the health service and electronic systems used in intensive care units

	Overall total	Uses electronic medical record and prescription system		p value
		No	Yes	
	N (%)	N (%)	N (%)	
Uses electronic medical records				-
No	15 (7.35)	-	-	
Yes	189 (92.65)	-	-	
Hospital type				
Others	1 (0.49)	0 (0.0)	1 (0.53)	0.002
Public (run by SO or similar third parties)	29 (14.22)	2 (13.33)	27 (14.29)	
Voluntary	37 (18.14)	1 (6.67)	36 (19.05)	
Public (state-run)	49 (24.02)	10 (66.67)	39 (20.63)	
Private	88 (43.14)	2 (13.33)	86 (45.50)	
ICU type				
Neonatal	3 (1.47)	0 (0.0)	3 (1.59)	0.257
Neurological	4 (1.96)	0 (0.0)	4 (2.12)	
Others	6 (2.94)	1 (6.67)	5 (2.65)	
Cardiological	7 (3.43)	2 (13.33)	5 (2.65)	
Mixed neonatal and pediatric	13 (6.37)	2 (13.33)	11 (5.82)	
Pediatric	35 (17.16)	2 (13.33)	33 (17.46)	
General Adult	136 (66.67)	8 (53.33)	128 (67.72)	
Electronic system				
Not used	15 (7.39)	15 (100.0)	0 (0.0)	< 0.001
Hospital e-SUS (substitute for HOSPUB)	2 (0.99)	0 (0.0)	2 (1.06)	
Other	31 (15.27)	0 (0.0)	31 (16.49)	
Private System B	31 (15.27)	0 (0.0)	31 (16.49)	
In-house system	45 (22.17)	0 (0.0)	45 (23.94)	
Private System A	79 (38.92)	0 (0.0)	80 (42.02)	
Length of system use (years)				
Not used	15 (7.35)	15 (100.0)	0 (0.0)	< 0.001
< 2	34 (16.67)	0 (0.0)	34 (17.99)	
2 - 4	52 (25.49)	0 (0.0)	52 (27.51)	
4 - 6	39 (19.12)	0 (0.0)	39 (20.63)	
6 - 8	21 (10.29)	0 (0.0)	21 (11.11)	
> 8	43 (21.08)	0 (0.0)	43 (22.75)	

SO - social organization; ICU - intensive care unit; SUS - *Sistema Único de Saúde*; HOSPUB - public hospital.

Among the participants who did not use EMRSs, 66.7% worked in state-run public hospitals, followed by private hospitals and public hospitals run by social organizations (13.3% each). As for those who used EMRSs, 45.5% worked in private hospitals, followed by state-run public hospitals (20.6%) and voluntary hospitals (19%). The p-value obtained in the Pearson chi-square test was 0.002, indicating that the distribution was not random and that the findings were statistically significant. The use of EMRSs was prevalent in private hospitals, and non-use was observed more frequently in state-run public hospitals.

There was no statistical significance in the sample distribution between the types of ICUs of the participating physicians, which is understandable considering that the type of hospital management had a greater influence on the implementation of the EMRS than the type of ICU involved. The majority of the participants worked in general adult ICUs; 53.3% of them did not use electronic systems, and 67.8% of them used EMRSs.

**Figure 1:**
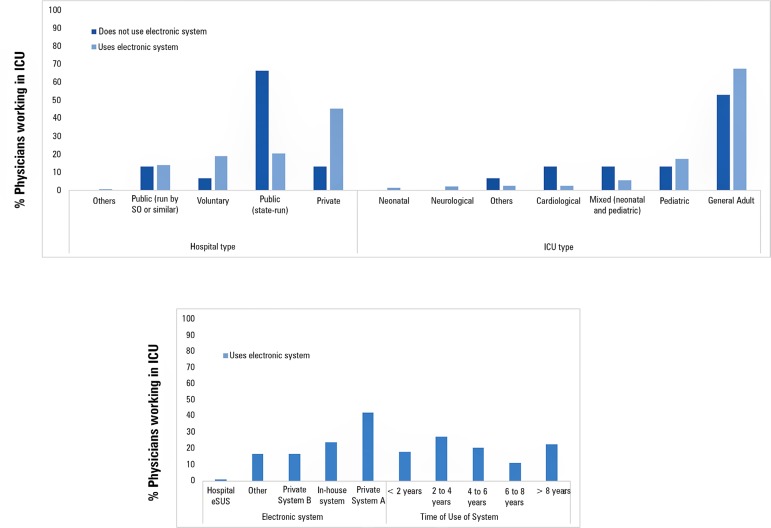
Profile of the use of electronic medical record and prescription systems. SO - social organization; ICU - intensive care unit; SUS - *Sistema Único de Saúde*.

Private System A was the most used (42%) system among physicians working in an ICU, followed by in-house electronic systems (24%) and Private System B (16.5%). Regarding the systems' implementation times in the hospitals, there was no predominance of a specific period of time, and a gradual and steady implementation of EMRSs could be observed in health care services since the 2000s. The results showed that 27.5% had used electronic systems in their ICUs for between 2 and 4 years, 22.7% for over 8 years and 20.6% for 4 to 6 years (Figure 1).

In regard to questions relating to quality improvements afforded by EMRSs, the majority (84.65%) of the participants believed that theirs had provided an improvement, and only 7.92% did not believe that the quality was superior to that of paper records. Comparing those who used electronic systems and those who did not, the electronic system users were more critical about quality. Among the physicians who did not use these tools, none believed that the quality could be inferior to that of paper records. In turn, 8.5% of EMRSs users considered them inferior to paper records (Table 2).

**Table 2:** Evaluation of safety and quality in the use of electronic medical record and prescription systems in intensive care units

	Overall total	Uses electronic medical record and prescription system		p value
		No	Yes	
	N (%)	N (%)	N (%)	
Affords greater quality				
No	16 (7.92)	0 (0.00)	16 (8.47)	0.549
Don’t know	15 (7.43)	1 (7.69)	14 (7.41)	
Yes	171 (84.65)	12 (92.31)	159 (84.13)	
Affords greater safety				
No	22 (10.89)	2 (15.38)	20 (10.58)	0.082
Don’t know	25 (12.38)	4 (30.77)	21 (11.11)	
Yes	155 (76.73)	7 (53.85)	148 (78.31)	

In regards to safety, 76.7% of the sample believed that their EMRS offered greater safety than paper systems, while 10.9% believed that it did not offer increased safety. A comparison of the groups of users and non-users of electronic systems revealed behavior contrary to what was shown in response to the quality questions: among non-users, 15.4% felt that there was greater safety, and among users, 10.6% were of the same opinion (Table 2).

The p-value found showed no significant difference and similar behavior between the groups. Both users and non-users rated the safety and quality of EMRSs as higher than those of paper systems. However, non-users may have had a tendency to overvalue EMRSs, which should be considered.

**Figure 2:**
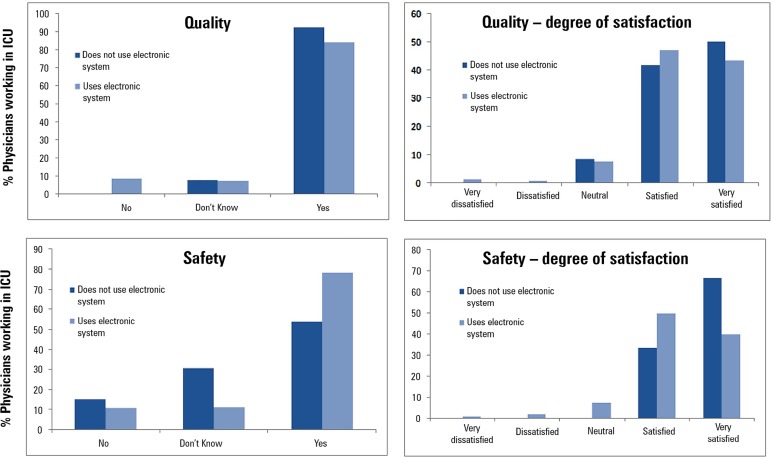
Perceptions of improvements in quality and safety in the use of electronic medical record and prescription systems. ICU - intensive care unit.

Physicians who believed that EMRSs offered superior safety compared to paper records were asked to rate the degrees of improvement/satisfaction related to those criteria, as shown in figure 2.

Table 3 shows the distribution of satisfaction levels among users and non-users of EMRSs in terms of the safety and quality items.

**Table 3:** Satisfaction level in relation to quality and safety in the use of electronic medical record and prescription systems compared to paper medical records in intensive care units

	Overall total	Uses electronic medical record and prescription system		Mean score (0 - 10)	Standard deviation
		No	Yes		
	N (%)	N (%)	N (%)		
Quality					
Very dissatisfied	2 (1.17)	0 (0.00)	2 (1.26)	8.38	1.56
Dissatisfied	1 (0.58)	0 (0.00)	1 (0.63)		
Neutral	13 (7.60)	1 (8.33)	12 (7.55)		
Satisfied	80 (46.78)	5 (41.67)	75 (47.17)		
Very satisfied	75 (43.86)	6 (50.00)	69 (43.40)		
Safety					
Very dissatisfied	1 (0.65)	0 (0.00)	1 (0.68)	8.20	1.52
Dissatisfied	3 (1.95)	0 (0.00)	3 (2.03)		
Neutral	11 (7.14)	0 (0.00)	11 (7.43)		
Satisfied	76 (49.35)	2 (33.33)	74 (50.00)		
Very satisfied	63 (40.91)	4 (66.67)	59 (39.86)		

The mean scores achieved, both for the quality (8.20) and safety (8.38) items, show a satisfactory degree of improvement when using EMRSs. Those who did not use EMRSs rated themselves as "very satisfied" (50.0% quality and 66.7% safety) more often than those who had used such systems (43.4% quality and 39.9% safety). However, regardless of the use or non-use of electronic systems, the perceptions of physicians working in ICUs who considered EMRSs to be safer and of better quality were satisfactory.

A quality and safety analysis, stratified by the electronic system, was also carried out to identify possible similarities and differences between the systems, as shown in table 4.

**Table 4:** Evaluation of safety and quality by electronic medical record and prescription system in intensive care units

	Private system A	In-house system	Private system B	e-SUS	Other	Not used	p value
	N (%)	N (%)	N (%)	N (%)	N (%)	N (%)	
Quality							
Very dissatisfied	0 (0.00)	2 (100.0)	0 (0.00)	0 (0.00)	0 (0.00)	0 (0.00)	0.345
Dissatisfied	1 (100.0)	0 (0.00)	0 (0.00)	0 (0.00)	0 (0.00)	0 (0.00)	
Neutral	3 (23.08)	4 (30.77)	4 (30.8)	0 (0.00)	1 (7.69)	1 (7.69)	
Satisfied	41 (51.25)	11 (13.75)	11 (13.8)	1 (1.25)	11 (13.8)	5 (6.25)	
Very satisfied	23 (30.67)	19 (25.33)	13 (17.3)	0 (0.00)	14 (18.7)	6 (8.00)	
Safety							
Very dissatisfied	0 (0.00)	1 (100.0)	0 (0.00)	0 (0.00)	0 (0.00)	0 (0.00)	0.811
Dissatisfied	0 (0.00)	1 (33.33)	1 (33.3)	0 (0.00)	1 (0.00)	0 (0.00))	
Neutral	4 (36.36)	3 (27.27)	3 (27.3)	0 (0.00)	1 (9.09)	0 (0.00)	
Satisfied	38 (50.0)	12 (15.79)	12 (15.8)	1 (1.32)	11 (14.5)	4 (2.63)	
Very satisfied	22 (34.92)	15 (23.81	8 (12.7)	1 (1.59)	13 (20.6)	2 (6.35)	

SUS - *Sistema Único de Saúde*.

With regard to quality, Private System A accounted for 30.7% of the very satisfied users and 51.2% of the satisfied users. This was followed by in-house system users, who accounted for 25.3% of the very satisfied users and 13.7% of the satisfied users. This distribution was also found in the safety item, with Private System A and in-house systems having the highest satisfaction percentages. Although we found no statistical significance in these specific results, it is interesting to note that in-house systems were, in a way, highly regarded by physicians in the ICUs.

## DISCUSSION

This was the first national study in the form of a questionnaire conducted among intensive care physicians on the use of EMRSs in Brazilian ICUs. The EMRS use rate was high (92.6%) among physicians who completed the questionnaire. This flies in the face of other publications, given that Brazil is considered a "developing country". A systematic review notes that "despite the great impact of information and communication technologies on clinical practice and on the quality of health services, this trend has been almost exclusive to developed countries, whereas countries with poor resources suffer from many economic and social issues that have hindered the real benefits of electronic health (eHealth) tools."^(10)^ Studies in different countries report different rates of EMRS use. In a review, Nguyen et al. note the increasing use of EMRSs around the world, from African and Latin American countries to the developed ones that have the highest use and growth rates.^(11)^ In the United States, increased adoption of EMRSs has been stimulated by the 2009 'meaningful use' initiative.^(12)^ An EMRS use rate of 39.1% has been reported in Spanish hospitals,^(13)^ while in Canada, EMRS adoption rates have increased from approximately 20% in 2006 to approximately 62% in 2013.^(14)^ Thus, the 92.6% use rate of EMRSs by physicians in Brazilian ICUs who completed the questionnaire indicates a high degree of computerization and use of IT resources.

Although greater adoption and growth of EMRS markets has been observed, EMRSs have demonstrated a surprising lack of benefits in evidence-based studies.

The perception of improved quality and safety when using EMRSs was high: 84.6% of physicians perceived improvements in quality and 76.7% perceived improvements in safety compared to paper records. Several studies in other countries have shown different use and satisfaction rates in regard to EMRSs in the contexts of different medical specialties. However, there is no evidence to suggest any improvement in patients' clinical outcomes due to the use of EMRSs. A systematic review and meta-analysis on the impact of EMRSs in ICUs showed no substantial effect on mortality, length of stay or cost.^(15)^ Despite the increased adoption and growth of EMRS markets, a surprising lack of benefits has been demonstrated in evidence-based studies of EMRSs.^(11)^

Unlike in other countries, this study showed a concentration of commercial EMRSs in the Brazilian market, with two systems (A and B) accounting for more than half of all of those used in ICUs (53.9%); these were followed by in-house systems (22.2%). The satisfaction levels of users of the different systems showed no significant difference for quality (p < 0.345) or safety (p = 0.811). The high amount of use of in-house systems raises some questions: (1) Is there dissatisfaction with the available commercial systems? (2) What is the cost effectiveness of commercial systems compared to the development of an in-house system? (3) Are in-house systems more or less safe than commercially available systems and is their quality higher or lower?

The attention of specialists in health care IT has been drawn to several issues not addressed in this study. The use of deficient systems and their misuse can cause errors that compromise the integrity of the information in EMRSs, leading to situations that present potential dangers and that affect patient safety or reduce health care quality.^(5)^ These unintended consequences can also increase cases of fraud and abuse and have serious legal implications.^(16)^ Moreover, a wide range of ethical, legal and technical issues currently prevents systematic entry of data into EMRSs and their use for clinical research purposes.^(17)^ In this regard, there is a tendency in the market towards system certification in which various aspects of safety and quality are evaluated.^(18)^

This study has some limitations. The lack of a demographic analysis of the respondents precludes generalization of the findings; we do not know if the physicians who responded to the questionnaire were more concentrated in one region of the country or if they were distributed equally throughout Brazil. This means that we do not know if the sample is homogeneous. Another important issue is related to the data collection method used for this study, in that it did not offer space for possible criticisms of EMRSs by the participants. For example, it is possible that a team could spend a lot of time filling in data in the EMRS at the expense of time at the patient's bedside, which is a possible safety issue. The number of respondents is consistent with the original intention, but the response rate was relatively low (4.3%). Without knowing if the sample is homogeneous, this sample size precludes generalizations of the results and external validation of the study. We cannot, therefore, establish broad conclusions based on this specific sample. Another important factor that was not included in the study was the cost of the implementation and maintenance of EMRSs; this would require a separate study.

Given the rate of use of EMRSs in Brazilian ICUs found in this study, a multicenter study focused on criticisms, possible safety issues and suggestions from EMRS users should be conducted to facilitate improvements to the systems that are currently in use.

## CONCLUSION

Electronic medical record systems seem to be widely used by intensive care physicians in Brazil. Although physicians reported relatively high satisfaction rates with electronic medical record systems, it is up to the information technology sector, scholars and medical assistants to work together to improve current systems in order to meet the needs of patients and health care professionals. As these new and innovative technological improvements emerge, this national study on the use of electronic medical record systems can serve as a basis for future comparisons and the evaluation of adoption and satisfaction rates, and it can provide a benchmark for future efforts in this rapidly evolving field.

### Author contributions

José Colleti Junior and Werther Brunow de Carvalho designed the study and wrote the discussion section. Alice Barone de Andrade analyzed the statistical data and contributed to the discussion section. José Colleti Junior wrote the final version of the manuscript.

## References

[1] Rojas CL, Seckman CA (2014). The informatics nurse specialist role in electronic health record
usability evaluation. Comput Inform Nurs.

[2] Wylie MC, Baier RR, Gardner RL (2014). Perceptions of electronic health record implementation: a
statewide survey of physicians in Rhode Island. Am J Med.

[3] Pageler NM, Longhurst CA, Wood M, Cornfield DN, Suermondt J, Sharek PJ (2014). Use of electronic medical record-enhanced checklist and
electronic dashboard to decrease CLABSIs. Pediatrics.

[4] Gillum RF (2013). From papyrus to the electronic tablet: a brief history of the
clinical medical record with lessons for the digital age. Am J Med.

[5] Wallace IM (2015). Is patient confidentiality compromised with the electronic health
record?: a position paper. Comput Inform Nurs.

[6] Carayon P, Wetterneck TB, Alyousef B, Brown RL, Cartmill RS, McGuire K (2015). Impact of electronic health record technology on the work and
workflow of physicians in the intensive care unit. Int J Med Inform.

[7] Perry JJ, Sutherland J, Symington C, Dorland K, Mansour M, Stiell IG (2014). Assessment of the impact on time to complete medical record using
an electronic medical record versus a paper record on emergency department
patients: a study. Emerg Med J.

[8] Ash JS, Sittig DF, Dykstra R, Campbell E, Guappone K (2009). The unintended consequences of computerized provider order entry:
findings from a mixed methods exploration. Int J Med Inform.

[9] Brown J (2011). Likert items and scales of measurement. Shiken: JALT Test Eval SIG News.

[10] Syzdykova A, Malta A, Zolfo M, Diro E, Oliveira JL (2017). Open-source electronic health record systems for low-resource
settings: systematic review. JMIR Med Inform.

[11] Nguyen L, Bellucci E, Nguyen LT (2014). Electronic health records implementation: an evaluation of
information system impact and contingency factors. Int J Med Inform.

[12] Adler-Milstein J, Everson J, Lee SY (2014). Sequencing of EHR adoption among US hospitals and the impact of
meaningful use. J Am Med Inform Assoc.

[13] Marca G, Perez A, Blanco-Garcia MG, Miravalles E, Soley P, Ortiga B (2014). The use of electronic health records in Spanish
hospitals. Health Inf Manag.

[14] (2015). Progress in electronic medical record adoption in
Canada. Can Fam Physician.

[15] Thompson G, O'Horo JC, Pickering BW, Herasevich V (2015). Impact of the electronic medical record on mortality, length of
stay, and cost in the hospital and ICU: a systematic review and
metaanalysis. Crit Care Med.

[16] Bowman S (2013). Impact of electronic health record systems on information
integrity: quality and safety implications. Perspect Health Inf Manag.

[17] Jensen PB, Jensen LJ, Brunak S (2012). Mining electronic health records: towards better research
applications and clinical care. Nat Rev Genet.

[18] Office of the National Coordinator for Health Information Technology
(ONC), Department of Health and Human Services (HHS) (2015). 2015. Edition Health Information Technology (Health IT) Certification
Criteria, 2015 Edition Base Electronic Health Record (EHR) Definition, and
ONC Health IT Certification Program Modifications. Final rule. Fed
Regist.

